# Fabrication Technology of Self-Dissolving Sodium Hyaluronate Gels Ultrafine Microneedles for Medical Applications with UV-Curing Gas-Permeable Mold

**DOI:** 10.3390/gels10010065

**Published:** 2024-01-15

**Authors:** Rio Yamagishi, Sayaka Miura, Kana Yabu, Mano Ando, Yuna Hachikubo, Yoshiyuki Yokoyama, Kaori Yasuda, Satoshi Takei

**Affiliations:** 1 Graduate School of Biotechnology and Pharmaceutical Engineering, Toyama Prefectural University, Imizu 939-0398, Toyama, Japan; rio.yamagishi@outlook.jp (R.Y.); sayaka13579@outlook.jp (S.M.); kyasuda@pu-toyama.ac.jp (K.Y.); 2 Department of Pharmaceutical Engineering, Toyama Prefectural University, Imizu 939-0398, Toyama, Japan; u018033@st.pu-toyama.ac.jp (K.Y.); u018001@st.pu-toyama.ac.jp (M.A.); u018024@st.pu-toyama.ac.jp (Y.H.); 3 Toyama Industrial Technology Research and Development Center, Takaoka 933-0981, Toyama, Japan; yokoyama@itc.pref.toyama.jp

**Keywords:** ultrafine microneedles, sodium hyaluronate, functional gels, transdermal drug delivery system, gas-permeable mold, nanoimprint lithography

## Abstract

Microneedles are of great interest in diverse fields, including cosmetics, drug delivery systems, chromatography, and biological sensing for disease diagnosis. Self-dissolving ultrafine microneedles of pure sodium hyaluronate hydrogels were fabricated using a UV-curing TiO_2_-SiO_2_ gas-permeable mold polymerized by sol-gel hydrolysis reactions in nanoimprint lithography processes under refrigeration at 5 °C, where thermal decomposition of microneedle components can be avoided. The moldability, strength, and dissolution behavior of sodium hyaluronate hydrogels with different molecular weights were compared to evaluate the suitability of ultrafine microneedles with a bottom diameter of 40 μm and a height of 80 μm. The appropriate molecular weight range and formulation of pure sodium hyaluronate hydrogels were found to control the dissolution behavior of self-dissolving ultrafine microneedles while maintaining the moldability and strength of the microneedles. This fabrication technology of ultrafine microneedles expands their possibilities as a next-generation technique for bioactive gels for controlling the blood levels of drugs and avoiding pain during administration.

## 1. Introduction

Recent advancements in nanotechnology have ushered in a paradigm shift in microneedle manufacturing techniques [1]. Microneedles have attracted substantial interest across diverse domains, including cosmetics [2,3], drug delivery systems (DDS) [4,5], and biological sensing for disease diagnosis [6,7]. For example, the cosmetics industry is leveraging microneedle technology to treat skin wrinkles and is developing several solid and dissolvable microneedle-based products incorporating anti-wrinkle formulations [8,9]. In the pharmaceutical industry, the development of dissolvable microneedle patches for insulin [10,11] and vaccines [12] is expected. In the area of sensing applications, wearable microneedle sensor arrays for continuous minimally invasive transdermal analysis of drugs of abuse and nerve agents are being developed in the medical field [13], and microneedle sensor arrays for food spoilage detection are being developed in the food field [14].

Microneedles are meticulously crafted, minimally invasive, and virtually painless micro-sized fine needles adept at breaching the stratum corneum barrier, thus emerging as prime candidates for achieving systemic effects through transdermal delivery [15]. In the past two decades, significant progress has been made in the development of microneedle-based DDS. These systems temporarily disrupt the superficial layers of the skin through diffusion [16,17]. Microneedle formulations encompass various categories defined by their manufacturing methods, including solid microneedles, hollow microneedles, coated microneedles, and self-dissolving microneedles [18,19,20]. However, solid, hollow, and coated microneedles, which are molded using materials such as silicone, stainless steel, and titanium, may pose a risk of needle breakage and residue in the skin, as well as the generation of sharp needle waste that is biologically harmful [21,22]. Particularly, silicone-based microneedles are fragile and can easily deteriorate, leaving residues on the skin and possibly eliciting an immune response [23]. Self-dissolving microneedles are perceived as the most effective solution for ameliorating the deficiencies of other microneedle types [24]. Self-dissolving microneedles markedly enhance the drug loading capacity by encapsulating the drug throughout the needle rather than just on the outer surface [25,26]. Furthermore, because the microneedle dissolves during drug delivery, there is no risk of the needle remaining in the body, thus eliminating the peril of needle waste, which is a biohazard [27]. In particular, research on the physicochemical properties of hyaluronic acid gel and its effects on the human body has made hyaluronic acid an ideal biomaterial for self-dissolving microneedles [28]. 

Conventional microneedles generally have heights of 100–1000 µm [29]. The length is related to its specific functions and applications. Wei Li et al. demonstrated that hormonal drug administration using self-dissolving microneedles with a matrix of a mixed solution of polyvinyl alcohol and sucrose showed relatively high delivery efficiency at a length of 800 μm [30]. In addition, Hongyao Du et al. demonstrated the potential of microneedles as an alternative treatment for psoriasis by fabricating microneedles with a height of 650 μm using hyaluronic acid as a matrix and containing methotrexate [31]. Microneedles with lengths of 100–1000 µm are considered to enable efficient drug delivery throughout the body. However, the shorter the needle, the less pain there will be, and the increase in drug concentration in the blood may be more gradual. New possibilities for transdermal administration using ultrafine microneedles of 100 μm or less are expected.

**Figure 1:**
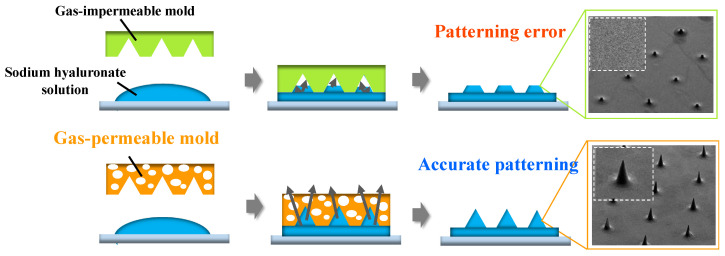
Comparison of nanoimprint processes by using gas-impermeable mold and gas-permeable mold.

In terms of microneedle manufacturing methods, microelectromechanical systems (MEMS) are used to mold hollow and dissolvable microneedles [32]. Metal microneedles have been manufactured using 3D laser cutting [33] or laser ablation [34]. Dissolvable microneedles were fabricated from computer-aided design (CAD) files using droplet-born air blowing (DAB), a droplet-sized microneedle polymer formed by an air-blowing process [35,36], continuous liquid interface manufacturing (CLIP), additive manufacturing (AM), three-dimensional (3D) printing, and micro-molding methods [37,38,39]. The micro-molding method has no theoretical limit on resolution because the mold can be designed and is one of the most suitable technologies for manufacturing ultrafine microneedles [40,41]. Nanoimprint lithography is a type of micro-molding method. As shown in Figure 1, in conventional nanoimprint processes, which use quartz or metal molds, the high interfacial tension between the solution and mold constrains the complete filling of the mold with the drug, leading to insufficient tip sharpness owing to the presence of bubbles [42]. Therefore, in the case of hyaluronic acid, which has a high molecular weight and high viscosity, bubbles are easily trapped in the mold, making it difficult to achieve ultrafine processing of 100 μm or less.

We previously reported the fabrication of self-dissolving hyaluronic acid ultrafine microneedles under heating conditions using nanoimprint lithography, a form of micro-molding that employs a heat-curing TiO_2_-SiO_2_ gas-permeable mold [43]. Gas-permeable molds could improve the molding defects caused by trapped gas by making the molds porous [44,45]. However, since this method requires vacuum drying at 40 °C, there were concerns that the drug would decompose, especially for heat-sensitive substances such as insulin and vaccines [46]. Peptide formulations require continuous cold storage and transportation (≤4 °C) [47]. Therefore, it was difficult for these microneedles to encapsulate these drugs with previously developed ultrafine microneedles. Additionally, owing to rapid drying due to heating, the area that could be manufactured without molding defects became smaller.

In this study, we developed an ultraviolet (UV)-curing TiO_2_-SiO_2_ gas-permeable mold, achieved through molecular design, and successfully improved gas permeability. Subsequently, we identified fabricating conditions that enable self-dissolving sodium hyaluronate ultrafine microneedles with varying molecular weights with a bottom diameter of 40 μm and a height of 80 μm at a low temperature of 5 °C, taking into account heat-unstable drugs. We compared the strength, puncture properties, and dissolution behavior of sodium hyaluronate gels with different molecular weights and evaluated the suitability of self-dissolving ultrafine microneedles as a base material for transdermal preparations.

## 2. Results and Discussion

### 2.1. Oxygen Gas Permeability Measurement Test

**Figure 2:**
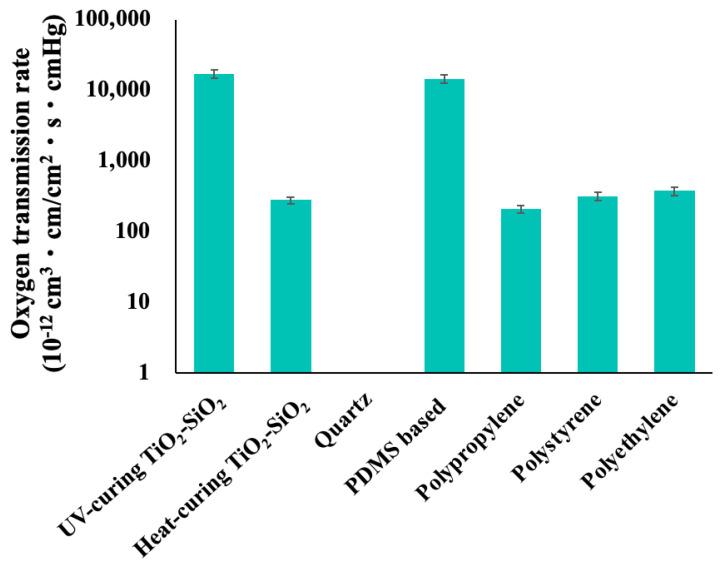
Results of oxygen gas permeability of UV-curing TiO_2_-SiO_2_ gas-permeable material, heat-curing TiO_2_-SiO_2_ gas-permeable material, quartz, poly (dimethylsiloxane), polypropylene, polystyrene, and polyethylene.

Figure 2 shows the results of the oxygen gas permeability measurement test. The oxygen gas permeability, expressed as 10^−12^ cm^3^·cm/cm^2^·s·cmHg, of UV-curing TiO_2_-SiO_2_ gas-permeable material, heat-curing TiO_2_-SiO_2_ gas-permeable material, quartz, poly (dimethylsiloxane) (PDMS)-based gas-permeable material, polypropylene, polystyrene, and polyethylene were 17,200, 290, 0, 14,800, 210, 320, and 380, respectively. The oxygen gas permeability of the UV-curing TiO_2_-SiO_2_ gas-permeable material, guided by a meticulously designed molecular architecture, significantly surpassed that of the conventional heat-curing TiO_2_-SiO_2_ gas-permeable material. In conventional heat-curing TiO_2_-SiO_2_ gas-permeable materials, concerns have arisen regarding polymer shrinkage during the curing process, which is attributable to the condensation reaction between the silanol group (Si-OH) generated during sol-gel polymerization and the SiH group present in the cross-linking agent and polymer. Conversely, the UV-curing TiO_2_-SiO_2_ gas-permeable material is expected to have smaller shrinkage and sparser crosslink density than the heat-curing TiO_2_-SiO_2_ gas-permeable material, contributing to a significant improvement in gas permeability.

These gas permeability values clearly outperformed those of three commonly used general-purpose plastics: polypropylene, polystyrene, and polyethylene. Furthermore, the gas permeability of the UV-curing TiO_2_-SiO_2_ gas-permeable material was comparable to that of PDMS-based gas-permeable material [48], a well-established polymer known for its high gas permeability. Notably, the UV-curing TiO_2_-SiO_2_ gas-permeable material, in contrast to the PDMS-based gas-permeable material, which relies solely on silicon, incorporates titanium along with silicon. This strategic inclusion enables control over the flexibility and strength of the mold. Additionally, the PDMS-based gas-permeable material required curing at 80 °C for 30 min [49], while the UV-curing TiO_2_-SiO_2_ gas-permeable material achieved complete curing within a mere 2 min, significantly expediting the mold manufacturing process. These results validated the efficacy of the UV-curing variant as a TiO_2_-SiO_2_ gas-permeable material.

### 2.2. Patterning of Sodium Hyaluronate Ultrafine Microneedles Using UV-Curing TiO_2_-SiO_2_ Gas-Permeable Mold

**Figure 3:**
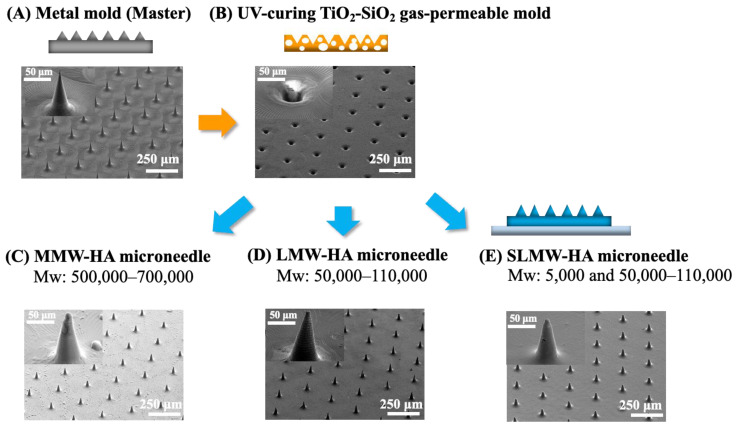
SEM images of the mold surfaces and ultrafine microneedle patterning structures: (**A**) The metal mold (master), (**B**) UV-curing TiO_2_-SiO_2_ gas-permeable mold, (**C**) MMW-HA ultrafine microneedle, (**D**) LMW-HA ultrafine microneedle, and (**E**) SLMW-HA ultrafine microneedle.

Figure 3 shows the scanning electron microscope (SEM) images of the mold surfaces and ultrafine microneedle patterning structures. (A) Metal mold (master), (B) UV-curing TiO_2_-SiO_2_ gas-permeable mold fabricated using (A), (C) MMW-HA (sodium hyaluronate with a molecular weight of 500,000–700,000) ultrafine microneedle, (D) LMW-HA (sodium hyaluronate with a molecular weight of 50,000–110,000) ultrafine microneedle, and (E) SLMW-HA (sodium hyaluronate mixed with molecular weights of 5000 and 50,000–110,000) ultrafine microneedle.

Prior studies have underscored the molding limitations associated with hyaluronic acid microneedles when employing quartz or metal molds with gas impermeability. These limitations include molding defects stemming from the high interfacial tension between the solution and mold as well as the entrapment of bubbles at the needle tips [50,51]. Conversely, the utilization of the UV-curing TiO_2_-SiO_2_ gas-permeable mold ameliorates these issues by expelling bubbles through its porous structure and enhancing the overall mold quality. In this study, as evident from the results in (C), (D), and (E), the use of the UV-curing TiO_2_-SiO_2_ gas-permeable mold unequivocally enabled the fabrication of ultrafine microneedles with sharp tips. Notably, this obviated the need for conventional mold-filling procedures such as centrifugation and vacuuming.

In the past, ultrafine microneedle molding using the heat-curing TiO_2_-SiO_2_ gas-permeable mold required vacuum drying at 40 °C, thereby impeding the sealing of heat-sensitive drugs. Furthermore, the available moldable area was restricted to approximately half the size of the mold owing to rapid drying during heating. The innovative UV-curing TiO_2_-SiO_2_ gas-permeable mold developed in this study substantially enhanced gas permeability, facilitating the fabrication of ultrafine microneedles under a low-temperature setting of 5 °C, which is a measure taken to safeguard heat-sensitive drugs.

In addition, it is generally considered more difficult to fabricate ultrafine microneedles with large-molecular-weight sodium hyaluronate because of their high viscosity. The excellent functionality of the UV-curing TiO_2_-SiO_2_ gas-permeable mold enabled the successful fabrication of sodium hyaluronate ultrafine microneedles with a molecular weight of 500,000–700,000 and a bottom diameter of 40 μm and a height of 80 μm.

### 2.3. Mechanical Strength Measurement Test

**Figure 4:**
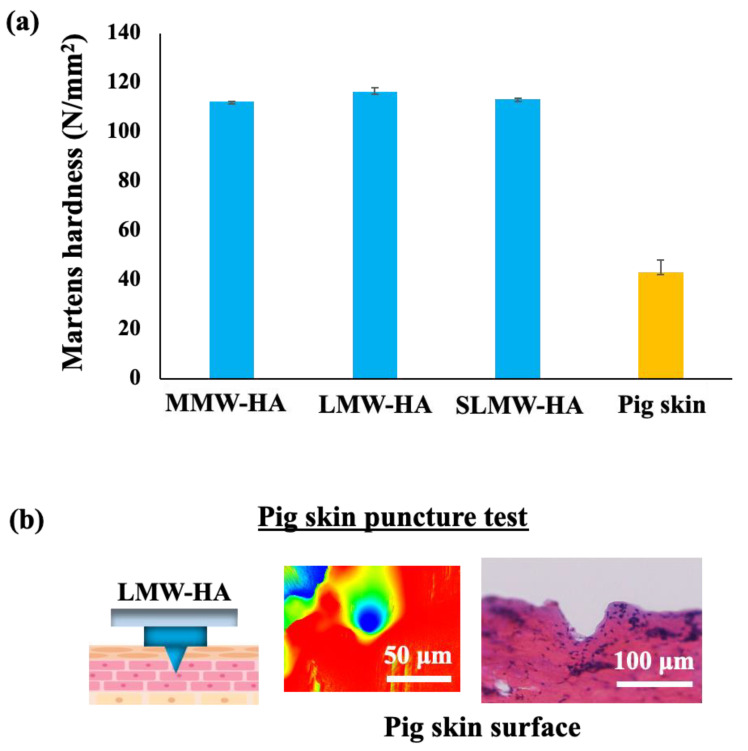
(**a**) Martens hardness of MMW-HA, LMW-HA, SLMW-HA, and pig skin, and (**b**) confocal microscope and frozen section images of pig skin surface after LMW-HA ultrafine microneedle puncture.

Figure 4a shows the results of the mechanical strength measurement test using a nanoindenter. The Martens hardness values for MMW-HA, LMW-HA, SLMW-HA, and pig skin surfaces were 116, 112, 113, and 43.2 N/mm^2^, respectively. The strength values of all sodium hyaluronates surpassed those of the pig skin surface, which closely resembles human skin. This underscores the robustness of sodium hyaluronate ultrafine microneedles for puncturing the skin. Remarkably, the strength values remained relatively consistent, irrespective of the molecular weight of sodium hyaluronate. Prior research has suggested that hyaluronic acid strength diminishes with increasing molecular weight due to heightened molecular aggregation and structural deformations in sodium hyaluronate with a high molecular weight. Such inefficiencies in molecular packing subsequently reduce mechanical strength [52]. Nevertheless, the findings in Figure 4 reveal comparable mechanical strengths across the molecular weight bands of the three sodium hyaluronates assessed in this study. It is generally known that the lower the molecular weight of hyaluronic acid, the lower the film-forming ability of hyaluronic acid. However, it was found that adding 27 wt% sodium hyaluronate with a molecular weight of 50,000–110,000 to sodium hyaluronate with a molecular weight of 5000 can improve the strength of ultrafine microneedles.

In an additional pig skin puncture test, shown in Figure 4b, traces of LMW-HA ultrafine microneedles were observed on the skin surface using a confocal microscope. Similarly, LMW-HA ultrafine microneedle traces were observed on the surface of pig skin when frozen sections were obtained after puncturing the skin. Therefore, it was revealed that the LMW-HA ultrafine microneedle had sufficient strength for puncturing pig skin. Based on this result, it was considered that the other two types of microneedles with equivalent strengths have sufficient strength to puncture the pig skin.

### 2.4. Dissolution Behavior of Sodium Hyaluronate Ultrafine Microneedles with Different Molecular Weights

**Figure 5:**
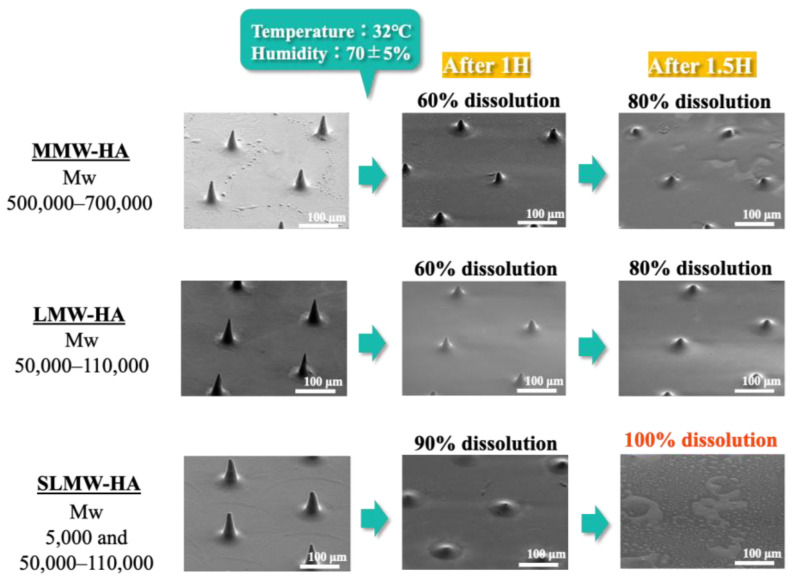
Results of the test of dissolution behavior of sodium hyaluronate ultrafine microneedles with different molecular weights by environmental test apparatus.

Figure 5 shows the dissolution behavior of the sodium hyaluronate ultrafine microneedles. The sodium hyaluronate ultrafine microneedle with SLMW-HA achieved 90% dissolution within 1 h under the specified temperature and humidity conditions, culminating in complete dissolution after 1.5 h. In contrast, sodium hyaluronate ultrafine microneedles with MMW-HA and those with LMW-HA achieved approximately 60% dissolution after 1 h and 80% dissolution after 1.5 h. Combined with the aforementioned mechanical strength measurement results, the results demonstrate that adding sodium hyaluronate with a molecular weight of 50,000–110,000 to sodium hyaluronate with a molecular weight of 5000 can improve membrane strength owing to the properties of a high molecular weight while achieving the excellent solubility of sodium hyaluronate with a molecular weight of 5000. Notably, no significant difference was observed in the dissolution behavior of MMW-HA and LMW-HA ultrafine microneedles. Typically, hyaluronic acid with a higher molecular weight takes longer to dissolve. However, the ultrafine microneedles employed in this study, characterized by a bottom diameter of 40 µm and a height of 80 µm, are finer than the conventional microneedles, which typically have a bottom diameter of 50–250 µm and a height of 100–1000 µm. This distinction can be attributed to the amplified dissolution rate associated with the larger surface area.

The choice of the base material has a significant impact on the transdermal permeability of drugs. Various drugs administered transdermally via microneedles exhibited different optimal permeation rates. The results of the dissolution behavior studies indicate that ultrafine microneedles have the potential to be useful base materials for setting the appropriate solubility for each transdermally administered drug by optimizing the molecular weight formulation of sodium hyaluronate.

## 3. Conclusions

By leveraging nanoimprint lithography with the newly devised UV-curing TiO_2_-SiO_2_ gas-permeable mold, characterized by a porous structure, we identified the optimal molding conditions for fabricating self-dissolving sodium hyaluronate ultrafine microneedles with diverse molecular weights at a low temperature of 5 °C, a measure that accounts for heat-sensitive drug considerations. In addition, the previously reported heat-curing TiO_2_-SiO_2_ gas-permeable material and PDMS-based gas-permeable material, an established gas-permeable mold, require elevated temperatures of approximately 80−180 °C and prolonged cross-linking durations of approximately 20−30 min for polymer curing. In stark contrast, the UV-curing variant enabled rapid curing for 2 min at room temperature using judicious molecular design. Consequently, the manufacturing time for the gas-permeable mold was substantially reduced, thereby augmenting the overall throughput. Furthermore, the moldability, strength, and solubility of three types of sodium hyaluronate with varying molecular weights were evaluated, confirming their suitability as microneedles. It was demonstrated that the UV-curing TiO_2_-SiO_2_ gas-permeable mold can be used to mold microneedles regardless of the molecular weight of the hyaluronic acid. In addition, mechanical strength measurements of the three types of sodium hyaluronate showed Martens hardness values higher than those of pig skin, and puncture tests showed that the ultrafine microneedles were strong enough to penetrate pig skin.

The dissolution behavior varied depending on the molecular weight of sodium hyaluronate. In particular, it was found that adding 27 wt% sodium hyaluronate with a molecular weight of 50,000–110,000 to sodium hyaluronate with a molecular weight of 5000 may result in both excellent mechanical strength and excellent solubility.

These results indicate that ultrafine microneedles may be a useful substrate for optimizing the molecular weight formulation of sodium hyaluronate and establishing the appropriate solubility for each transdermal drug, and a new function of ultrafine microneedles is that they are finer than conventional microneedles as a DDS.

## 4. Materials and Methods

### 4.1. Materials

#### 4.1.1. Materials for UV-Curing TiO_2_-SiO_2_ Gas-Permeable Mold

**Figure 6:**
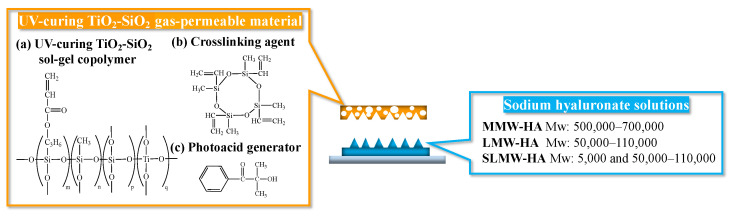
Molecular structure of material used to synthesize UV-curing TiO_2_-SiO_2_ gas-permeable mold and different molecular weight types of sodium hyaluronates.

The left side of Figure 6 shows the molecular structure of the material used to synthesize the UV-curing TiO_2_-SiO_2_ gas-permeable mold and different molecular weight types of sodium hyaluronates. The UV-curing TiO_2_-SiO_2_ gas-permeable mold was composed of four constituents: 40 wt% 3-(acryloyloxy)propyltrimethoxysilane, 35 wt% methyltrimethoxysilane, 15 wt% tetraethyl titanate, and 10 wt% tetraethoxysilane. This copolymer was synthesized via sol-gel polymerization (Figure 6a). Subsequently, 87 wt% of the sol-gel polymer was combined with 2,4,6,8-tetramethyl-2,4,6,8-tetravinylcyclotetrasiloxane (T2523, Tokyo Chemical Industry, Tokyo, Japan) (Figure 6b) as the cross-linking agent. A 3 wt% solution of 2-hydroxy-2-methyl-1-phenylpropanone (Omnirad 1173, Toyotsu Chemiplas, Tokyo, Japan) (Figure 6c) was added as the UV radical polymerization initiator. The mixture was stirred for 8 h using a mixing rotor (MR-5, Az One, Osaka, Japan) to obtain the UV-curing TiO_2_-SiO_2_ gas-permeable material.

The UV-curing TiO_2_-SiO_2_ gas-permeable material distinguishes itself by enabling rapid curing, which takes only approximately 2 min through molecular design, in contrast to conventional heat-curing TiO_2_-SiO_2_ gas-permeable materials, which require heating at 180 °C for 20 min of cross-linking to solidify the polymer. This is also an advantage over conventional PDMS-based gas-permeable materials that require curing at 80 °C for 30 min. This provides a significant advantage by reducing mold manufacturing time and enhancing throughput.

The characteristics of molds in nanoimprint lithography require both mechanical strength when the mold is pressed against the transfer material and flexibility when the mold is released after the transfer materials are cured. In gas-permeable molds, the gas permeation properties can be controlled by controlling the cross-linking density of the gas-permeable material through molecular design. A sparse crosslink density in a gas-permeable material increases the gas permeability but decreases the mechanical strength of the mold. In other words, there is a trade-off between gas permeability and mechanical strength. Our previous studies on heat-curing TiO_2_-SiO_2_ gas-permeable molds have shown that the inclusion of TiO_2_ by devising the molecular design improves the mechanical strength of gas-permeable molds compared to conventional SiO_2_-based gas-permeable molds, such as PDMS-based gas-permeable molds. The TiO_2_-SiO_2_ gas-permeable material, in contrast to the SiO_2_-based gas-permeable material, which relies solely on SiO_2_, incorporates TiO_2_ along with SiO_2_. This strategic inclusion enables control over the flexibility and strength of the gas-permeable mold. Therefore, the TiO_2_-SiO_2_ gas-permeable material has the advantage of being compatible with the trade-off characteristics of gas permeability and mechanical strength.

#### 4.1.2. Selection of Sodium Hyaluronate for Ultrafine Microneedles

The molecular weight of hyaluronic acid can have a significant effect on properties such as microneedle solubility, skin permeability, and mechanical strength. Hyaluronic acid is used in a wide range of molecular weights, ranging from several thousand to several million. Owing to their low mechanical strength and viscosity, low-molecular-weight hyaluronic acid with a molecular weight of less than 10,000 is rarely used as a material for microneedles. Hyaluronic acid with a high molecular weight of over 1,000,000 is not suitable for high-precision microneedle fabrication because its high viscosity is likely to cause incomplete filling when filling the molds [53]. In addition, high-molecular-weight hyaluronic acid with a molecular weight of over 1,000,000 may reduce the immunogenicity of the antigen and may not be suitable as a substrate for vaccination [54]. In contrast, the intermediate molecular weight range has various advantages, such as moderate viscosity, high mechanical properties, and ease of preparation. The penetration of hyaluronic acid into skin tissue differs according to molecular weight, with molecular weights of 10,000–250,000 allowing penetration into the dermal layer and 250,000–1,000,000 allowing penetration into the superficial layer. Different molecular weights also exhibit different biological properties. A molecular weight of 10,000–250,000 is used as the base material for treating tumors and for vaccination, whereas a molecular weight of 250,000–1,000,000 is used as the base material for insulin administration [23]. Considering these characteristics, three types of sodium hyaluronate were selected for this study: MMW-HA has a molecular weight of 500,000–700,000 (FCH-60, Kikkoman Biochemifa Company, Tokyo, Japan), LMW-HA has a molecular weight of 50,000–110,000 (FCH-SU, Kikkoman Biochemifa Company, Tokyo, Japan), and SLMW-HA has a mixture of molecular weights of 5000 (micro hyaluronic acid FCH, Kikkoman Biochemifa Company, Tokyo, Japan) and 50,000–110,000, as shown on the right side of Figure 6.

### 4.2. Methods

#### 4.2.1. Oxygen Gas Permeability Measurement Test

An oxygen gas permeability test was conducted on various materials, including UV-curing TiO_2_-SiO_2_ gas-permeable material, heat-curing TiO_2_-SiO_2_ gas-permeable material, quartz, PDMS-based gas-permeable material, polypropylene, polystyrene, and polyethylene. The measurements were performed using a differential pressure gas permeability measuring device (GTR-11, -11A, GTR Tec, Kyoto, Japan). Oxygen gas permeability was assessed under specific conditions, with the sample thickness measuring approximately 100 μm and the temperature maintained at 40–43 °C. Oxygen gas permeability was calculated by averaging the values obtained from the three measurements.

#### 4.2.2. Two-Step Nanoimprinting Process for Fabricating UV-Curing TiO_2_-SiO_2_ Gas-Permeable Mold and Sodium Hyaluronate Ultrafine Microneedles

**Figure 7:**
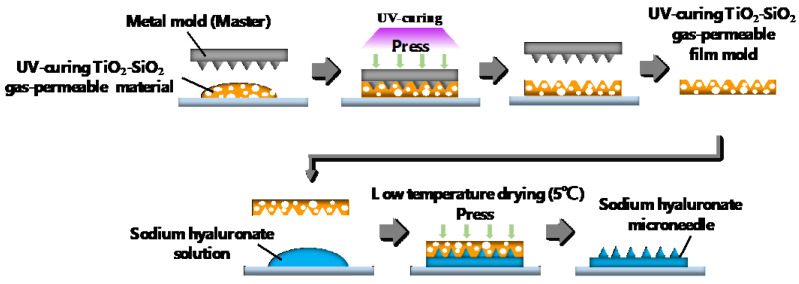
Double nanoimprint techniques used to transfer metal molds with master needle patterns to UV-curing TiO_2_-SiO_2_ gas-permeable molds to produce the transferred substrates with sodium hyaluronate ultrafine microneedle patterns.

Figure 7 shows the patterning processes of the UV-curing TiO_2_-SiO_2_ gas-permeable mold and sodium hyaluronate ultrafine microneedles with varying molecular weights. This study involves a two-step nanoimprinting process. First, the UV-curing TiO_2_-SiO_2_ gas-permeable material was imprinted using a metal master mold, which was then employed to imprint sodium hyaluronate with different molecular weights. Initially, a silane coupling agent (DURASURF DS-831TH, Harves, Saitama, Japan) was applied to the surface of a metal mold (20 mm × 20 mm) featuring needle patterns with a bottom diameter of 40 µm and a height of 80 µm. This surface treatment was allowed to dry for 2 h to facilitate the mold release. The UV-curing TiO_2_-SiO_2_ gas-permeable material was applied to a glass slide, and a metal master mold was placed at the top after mold release treatment and subjected to pressurization. UV irradiation was performed for 2 min using a UV spotlight source (Lightning Cure LC8, Hamamatsu Photonics, Shizuoka, Japan). The UV light intensity was measured using a UV illuminance meter (ACCU-CALTM-50, Dymax, Torrington, CT, USA), registered at 25 mW/cm^2^. The metal master mold was then removed, yielding the UV-curing TiO_2_-SiO_2_ gas-permeable mold with an inverted needle pattern. This mold, made in the form of a glass slide, exhibited easy detachment from the glass slide, enabling its independent use as a flexible-film-type gas-permeable mold.

Subsequently, water solutions of FCH-60 (5.50 wt% sodium hyaluronate), FCH-SU (14.6 wt% sodium hyaluronate), FCH (26.3 wt%), and FCH-SU (10.5 wt%) were prepared. Sodium hyaluronate was completely dissolved via ultrasonic treatment using an ultrasonic cleaner (OZL-2000, onezili). The sodium hyaluronate solutions were then defoamed using a centrifugal separator to remove air bubbles generated during dissolution. The glass substrate was subjected to hydrophilic treatment using UV ozone treatment equipment (LT0Z-180, Litho Tech Japan, Saitama, Japan) to enhance adhesion to sodium hyaluronate. The glass substrate was coated with the sodium hyaluronate solution, placed on a metal mold with the UV-curing TiO_2_-SiO_2_ gas-permeable mold, and subjected to pressurization. Three types of sodium hyaluronate ultrafine microneedles with distinct molecular weights were manufactured by drying under refrigerated conditions at 5 °C for 7 days and subsequently removing the UV-curing TiO_2_-SiO_2_ gas-permeable mold.

#### 4.2.3. Mechanical Strength Measurement Test

The Martens hardness of MMW-HA, LMW-HA, SLMW-HA, and pig skin surface (Dard Corporation, Tokyo, Japan) were gauged through indentation testing according to ISO 14577-1, utilizing a microhardness tester (Fischerscope^®^ HM2000, Fischer Instruments, Saitama, Japan). The elastic modulus was derived from the loading curves acquired during the nanoindentation tests performed three times under a 60 mN load.

#### 4.2.4. Pig Skin Puncture Test

A puncture test was conducted on the pig skin using an LMW-HA ultrafine microneedle. The LMW-HA ultrafine microneedle was punctured into the pig skin for 10 min, and the surface was observed using a confocal microscope (OPTELICS H1200, Laser Tec, Yokohama, Japan). The other puncture test was conducted by applying the LMW-HA ultrafine microneedle to the pig skin for 10 min, followed by HE staining of frozen sections and observation of skin cross-sections.

#### 4.2.5. Dissolution Behavior of Sodium Hyaluronate Ultrafine Microneedles with Different Molecular Weights

The dissolution behavior of the three types of sodium hyaluronate ultrafine microneedles, each with a different molecular weight, was assessed using an environmental test apparatus. The incubator (PIC-101, Az One, Osaka, Japan) was maintained at a temperature of 32 °C and a humidity of 70 ± 5%. Sodium hyaluronate ultrafine microneedles were extracted after 1 h and 1.5 h for each molecular weight, and the dissolution behavior was evaluated by observing alterations in shape. The solubility of each molecular weight of sodium hyaluronate ultrafine microneedles was calculated from the ratio of needle height before and after dissolution.

## Data Availability

The datasets generated and/or analyzed during the current study are not publicly available because they belong to ongoing research but are available from the corresponding author upon reasonable request.
